# Biomarkers Can Identify Pulmonary Tuberculosis in HIV-infected Drug Users Months Prior to Clinical Diagnosis

**DOI:** 10.1016/j.ebiom.2014.12.001

**Published:** 2014-12-02

**Authors:** Rosa Sloot, Maarten F. Schim van der Loeff, Erik W. van Zwet, Mariëlle C. Haks, Sytze T. Keizer, Maarten Scholing, Tom H.M. Ottenhoff, Martien W. Borgdorff, Simone A. Joosten

**Affiliations:** a Department of Clinical Epidemiology, Biostatistics and Bioinformatics, Academic Medical Center, University of Amsterdam, Amsterdam, The Netherlands; b Center for Infections and Immunity Amsterdam (CINIMA), Academic Medical Center, University of Amsterdam, Amsterdam, The Netherlands; c Department of Infectious Diseases, Public Health Service, Amsterdam, The Netherlands; d Department of Medical Statistics and Bioinformatics, Leiden University Medical Center, Leiden, The Netherlands; e Department of Infectious Diseases, Leiden University Medical Center, Leiden, The Netherlands; f Department of Medical Microbiology, Onze Lieve Vrouwe Gasthuis, Amsterdam, The Netherlands

**Keywords:** Tuberculosis, Host biomarkers, HIV, Gene expression, dcRT-MLPA

## Abstract

**Background:**

Current diagnostic tests cannot identify which infected individuals are at risk for progression to tuberculosis (TB). Our aim was to identify biomarkers which can predict the development of TB prior to clinical diagnosis.

**Method:**

In a retrospective case–control study, RNA of 14 HIV-infected drug users obtained before TB diagnosis (cases) and of 15 who did not develop TB (controls) was analyzed for the expression of 141 genes by dcRT-MLPA followed by Lasso regression analysis.

**Findings:**

A combined analysis of IL13 and AIRE had the highest discriminatory power to identify cases up to 8 months prior to clinical diagnosis. Cases expressing IL13 had a gene expression pattern strongly enriched for type I IFN related signaling genes, suggesting that these genes represent processes that contribute to TB pathogenesis.

**Interpretation:**

We here demonstrated that biomarkers, such as IL13-AIRE, can identify individuals that progress to TB within a high risk population, months prior to clinical diagnosis.

## Highlights

• A predictive signature was detected in ex vivo PBMCs of HIV-infected drug users up to 8 months prior to clinical TB diagnosis;
• Combined expression of IL13 and AIRE had the highest discriminatory power to identify cases months prior to TB diagnosis.
• Cases expressing IL13 had a gene expression pattern that was strongly enriched for type I IFN related signalling genes.

## 1 Introduction

The WHO estimates that there were 8.6 million cases of tuberculosis and 1.3 million deaths due to tuberculosis (TB) in 2012 (World Health Organization (WHO): Global Tuberculosis Report (2013)). Infection with *Mycobacterium tuberculosis* (*Mtb*) has an unpredictable outcome. The proportion of individuals who truly remain infected after tuberculin skin test (TST) or interferon gamma release assay (IGRA) conversion is unknown (Mack et al., 2009). An estimated 5–10% of infected individuals will develop pulmonary TB, mostly within the first 2 years following infection (Comstock et al., 1974, Vynnycky and Fine, 2000). At present it is impossible to predict who will progress to disease and who will remain latently infected.

Biomarkers (characteristics that can be objectively measured as indicators of a pathogenic process) (Biomarkers Definitions Working Group, 2001) have the potential to contribute significantly in the battle against *Mtb*. The vast majority of *Mtb*-infected individuals remain healthy, suggesting that immune responses in individuals that control latent infection with *Mtb* throughout their lifetime differ from responses in those who develop TB within the first years following infection (Weiner and Kaufmann, 2014, Schuetz et al., 2011). If true, correlates of TB progression should be identifiable, and these could be valuable in early identification of individuals at risk of developing – and transmitting – TB (Ottenhoff et al., 2012a). Furthermore, in low incidence countries biomarkers predictive of TB progression would be useful in selecting high-risk individuals for preventive therapy or regular screening. Examples are individuals with known or frequent exposure, such as contacts of infectious pulmonary TB patients or drug users. Likewise, TB-HIV co-infected individuals have an increased risk of progression to TB, reactivation of latent TB infection (LTBI) and TB related mortality (Nunn et al., 2005). Determination of such biomarkers could be embedded in existing programs in the Netherlands, in which HIV-infected individuals are actively screened for TB.

Several studies have identified host gene expression patterns in the blood from patients with pulmonary TB that differ from patterns in latently infected individuals or patients with other inflammatory conditions (Berry et al., 2010, Maertzdorf et al., 2011a, Maertzdorf et al., 2011b, Maertzdorf et al., 2012, Ottenhoff et al., 2012b, Cliff et al., 2013). Individual studies have mostly pointed towards type I interferon (IFN) signaling components as key markers of pulmonary TB, however, analysis of combined data indicated that besides type I IFN signaling also myeloid cell activation, general inflammation and B cell related markers are important players during TB (Joosten et al., 2013). To date no studies have identified biomarkers that can predict disease development well before the onset of clinical symptoms, and thus before the onset of contagious disease. The aim of this study was to explore whether biomarkers exist that may predict clinical TB. We used peripheral blood mononuclear cell (PBMC) samples of HIV-infected drug users, participating in a unique cohort, the Amsterdam Cohort Study (ACS), which undertook regular blood sampling and storage over several decades, independent from TB symptoms or diagnosis and thus allowed retrospective selection of samples prior to TB diagnosis.

## 2 Materials & Methods

### 2.1 Study Population

The Amsterdam Cohort Study (ACS) among drug users is an open and ongoing prospective cohort study, initiated in 1985 in Amsterdam, the Netherlands (van den Hoek et al., 1988). The ACS was approved by the medical ethics committee of the Academic Medical Center of Amsterdam. Regular users of hard drugs, such as heroin, cocaine or amphetamines, at least 3 times per week, were included in the ACS. Enrolment is voluntary and written informed consent was obtained from every participant at intake visit. Participants were asked to return for follow-up visits every 4 to 6 months at the Public Health Service (PHS). At every cohort visit, blood was drawn for laboratory testing and storage. Blood specimens have been tested for HIV-1 antibodies using consecutive generations of commercially available screening assays (obtained from Abbott Laboratories, Abbott Park, Illinois, USA; Organon International, The Netherlands, and bioMérieux, France) and confirmed by Western blot analyses (Genelabs Diagnostics, Singapore). Most of the participating drug users were also taking part in the methadone substitution program of the PHS Amsterdam. Methadone clients participating in the ACS were screened for tuberculosis at the TB department of the PHS Amsterdam by chest radiograph at six month intervals. A TB diagnosis was made by specialized TB clinicians, based on symptoms, chest radiograph, sputum smear and culture results, and/or by clinical response to treatment, based on national guidelines (KNCV Tuberculosis Foundation, 2008). In this cohort HIV infection increased the risk of tuberculosis 13-fold (Keizer et al., 2000). Since PBMCs were stored prospectively over decades, samples prior to clinical diagnosis of TB were available, and could be used to study gene expression profiles before the onset of TB.

### 2.2 Sample Selection

Twenty HIV-infected drug users diagnosed with TB in the period 1985–2012 were selected as cases using the ACS cohort database. A requirement was that at least one stored PBMC sample was available in the sample repository in the 365-day period preceding the date of the TB diagnosis. We preferred to select samples taken about 6 months before TB diagnosis, but we were limited in the number of samples available prior to TB diagnosis, as many samples from this cohort were already used for other research purposes. All TB cases with samples collected > 180 days before diagnosis were invited for at least 1 ACS cohort follow-up visit after the point of sample collection. Thirty HIV-infected drug users who were not diagnosed with TB in the same cohort period were selected as controls. The controls were frequency-matched for age, gender and CD4 cell count. At time of analysis, all controls were at least 4 years passed the PBMC collection time point and were not diagnosed with TB so far.

We checked the clinical files of the TB department to obtain details on TB diagnosis. In the process it was found that one case was diagnosed with LTBI (and not TB) at a time point shortly after sample collection; he developed TB 8 years later. Therefore this person was excluded from the analysis. Among the remaining 49 samples, from 29 (14 cases and 15 controls) sufficient good quality RNA was isolated for analysis. The 29 participants with sufficient RNA in their samples did not differ significantly from the other 20, in terms of age, gender, CD4 cell count, calendar period of sample, and TB status (case or control). Among the 29 included participants, cases did not differ significantly from controls in these respects (Table 1). The majority of cases were classified as PTB (57%) or PTB + EPTB (29%) and diagnosis was based on culture (86%) or clinical response to treatment (14%) (Table 1). There were no previous episodes of TB reported for any of the participants, cases or controls.

### 2.3 RNA Extraction

PBMCs were thawed using 50% FCS (Greiner Bio-One, Alphen aan den Rijn, the Netherlands), washed in PBS and the pellet was directly dissolved in 1 ml Trizol (Invitrogen, Thermo Fisher Scientific Inc., Merelbeke, Belgium). Total RNA was extracted according to standard protocols recommended by the manufacturer. RNA was quantified using a Nanodrop ND-1000 spectrophotometer and diluted to 50 ng/μl for use in dcRT-MLPA.

### 2.4 dcRT-MLPA Assay, Data Analysis and Quality Control

A dual-color reverse transcriptase multiplex ligation-dependent probe amplification (dcRT-MLPA) assay was performed as described previously (Joosten et al., 2012). Briefly, for each target-specific sequence, a specific RT primer was designed, located immediately downstream of the left and right hand half-probe target sequence. Following reverse transcription of 125 ng RNA using MMLV reverse transcriptase (Promega, Leiden, the Netherlands), left and right hand half-probes were hybridized to the cDNA at 60 °C overnight. Annealed half-probes were ligated and subsequently amplified by PCR (33 cycles of 30 s at 95 °C, 30 s at 58 °C and 60 s at 72 °C, followed by 1 cycle of 20 min at 72 °C). Primers and probes were from Sigma-Aldrich Chemie (Zwijndrecht, the Netherlands) (Joosten et al., 2012, Geluk et al., 2014) and MLPA reagents from MRC-Holland (Amsterdam, the Netherlands). PCR amplification products were 1:10 diluted in HiDi formamide containing 400HD ROX size standard and analyzed on an Applied Biosystems 3730 capillary sequencer in GeneScan mode (BaseClear, Leiden, the Netherlands).

Trace data were analyzed using the GeneMapper software package (Applied Biosystems). Signals below the threshold value for noise cut-off in GeneMapper (peak area below log^2^(200) = 7.64) were assigned the threshold value for noise cut-off. Subsequently, results from target genes were normalized to the average signal of housekeeping gene GAPDH and assigned the threshold value if below 7.64.

### 2.5 Statistical Analysis

Gene expression levels among cases and controls were plotted after log^2^ normalization and differences between cases and controls were compared using Mann–Whitney U testing; differences in expression were considered significant when P < 0.05. P-values for each individual comparison were corrected for multiple testing using the Benjamini–Hochberg adjustment (Benjamini and Hochberg, 1995). All gene expression data obtained using dcRT-MLPA were analyzed using Lasso regression analysis to identify the combined biomarkers with the highest discriminative power between cases and controls (Tibshirani, 1996). Lasso regression analysis is a shrinkage and selection method for linear regression that minimizes the usual sum of squared errors, with a bound on the sum of the absolute values of the coefficients (Tibshirani, 1996). Lasso has been previously used to identify biomarker signatures with the best predictive value between TB cases and individuals with LTBI (Joosten et al., 2012). The classifying capability of the biomarkers signatures is displayed as a receiver operating characteristics(ROC) curve, in which the true-positive rate (sensitivity) is plotted against the false positive rate (1 — specificity). The relationship between gene expression and time to TB diagnosis was assessed using linear regression analysis.

Molecular interactions between statistically significantly differently expressed genes were integrated using ‘Ingenuity Pathway Analysis’ (IPA) (www.ingenuity.com). Results were visualized as networks and ranked as canonical pathways. All analyses were completed in SPSS 21.0 (SPSS, Chicago, IL), GraphPad Prism (6.02, Graphpad Software Inc., La Jolla, Ca), and statistical program R version 2.11.0 (R Foundation, Vienna, Austria).

## 3 Results

### 3.1 Cases Can Be Identified Prior to Diagnosis Based on Gene Expression Patterns

**Fig. 1:**
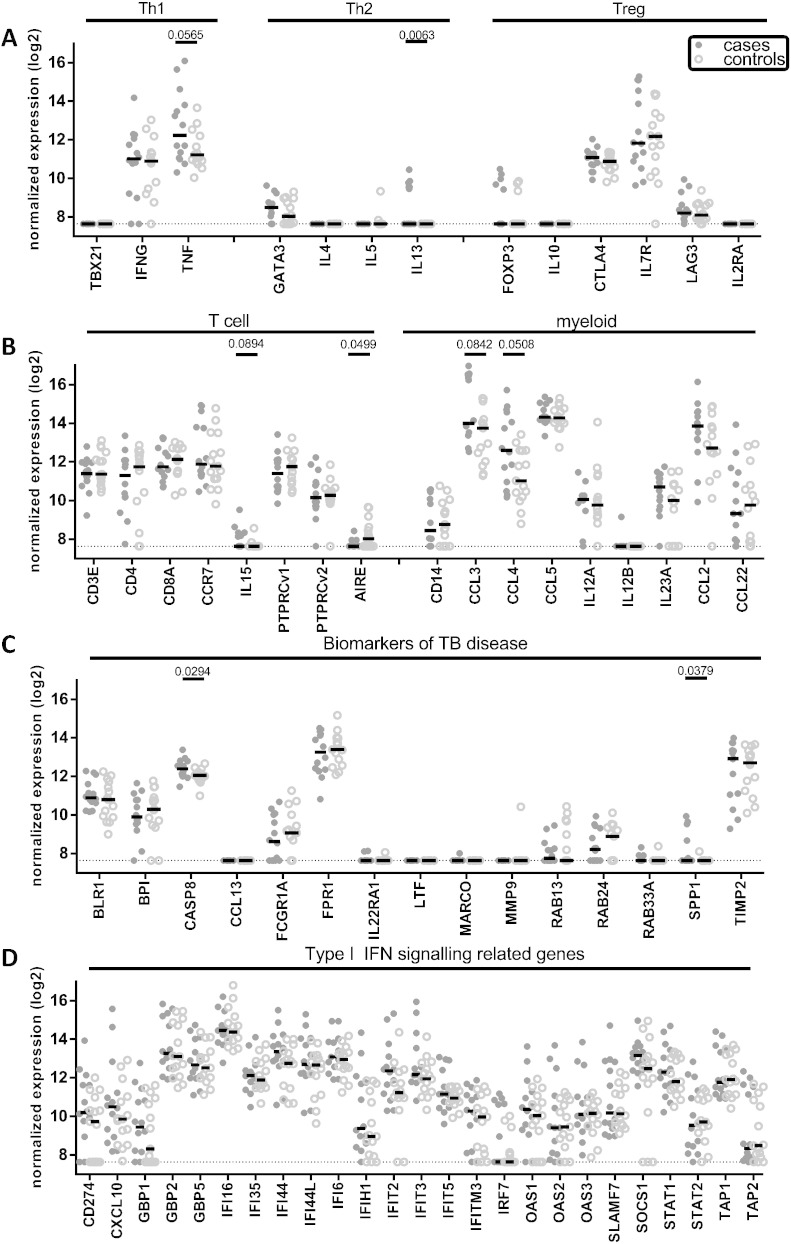
Differential gene expression pattern prior to TB diagnosis in cases versus controls. RNA was isolated from total PBMCs and gene expression levels were determined using dcRT-MLPA. Gene expression levels were normalized to the housekeeping gene GAPDH and log2 transformed before plotting. Cases (closed circles) and controls (open circles) were compared using Mann–Whitney testing and P values were shown for genes P < 0.1. A. Genes associated with Th1, Th2 and Treg function. B. Genes associated with general T-cell characteristics and genes associated with myeloid cell function. C. Genes previously reported to be important biomarkers in patients with TB disease at time of diagnosis. D. Genes belonging to the type I interferon signaling pathway, a pathway demonstrated to be highly active during active TB disease.

Expression patterns of human innate and adaptive immune response genes and genes known to be associated with TB were determined in samples from 14 cases prior to TB diagnosis and samples of 15 controls. Gene expression levels among cases and controls for a selection of the 141 included genes are presented in scatter plots (Fig. 1). Included were genes significantly differently expressed between cases and controls, and genes associated with known cellular responses. Fig. 1A depicts genes known to play a role in Th1, Th2 and regulatory T cell (Treg) associated immune responses. Generally these genes were expressed at equal levels in cases and controls, however the Th2 related gene IL13 (encoding interleukin 13) showed significantly increased expression in cases compared to controls. Significant IL13 gene expression was observed in 6/14 cases but in none of the controls. Fig. 1B includes genes expressed during general T cell development and genes associated with myeloid lineages. AIRE (APECED, autoimmune regulator) showed a significantly increased expression in controls compared to cases. Moreover, expression of the chemokine CCL4 (MIP1β) was increased in cases compared to controls (P = 0.051). In Fig. 1C a series of genes previously identified as biomarkers of active TB is shown; CASP8 (encoding caspase 8) and SPP1 (Secreted Phosphoprotein 1) showed a significant increase in cases compared to controls. Finally, Fig. 1D contains genes associated with type I IFN signaling, of which many have been previously identified as markers of active TB (Berry et al., 2010, Maertzdorf et al., 2011a, Maertzdorf et al., 2011b, Maertzdorf et al., 2012, Ottenhoff et al., 2012b, Cliff et al., 2013). None of these genes were expressed significantly differently between cases and controls. P values for the comparison of expression between cases and controls for all 141 genes are depicted in Fig. 4A. After adjusting for multiple testing, none of the genes remained significantly differentially expressed between cases and controls.

**Fig. 2:**
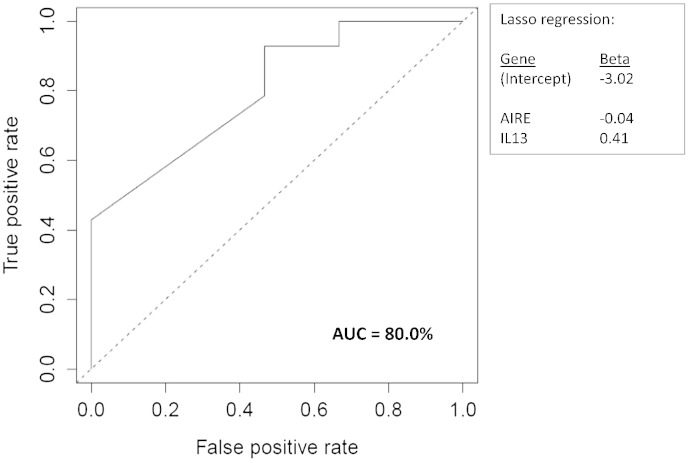
IL13 and AIRE are biomarkers discriminating cases and controls. All gene expression data obtained using dcRT-MLPA were analyzed using Lasso regression analysis to identify the biomarkers that contribute to the discrimination between cases and controls. Receiver operator characteristic (ROC) curve shows discriminative power of combined signature of IL13 and AIRE for the prediction of TB disease development.

Lasso regression analysis was used to determine biomarker signatures with the highest discriminatory power. The biomarker signature that classified controls versus cases included 2 genes, AIRE, with increased expression in controls (β, − 0.04) and IL13, with increased expression in cases (β, 0.41). The classifying value of these biomarkers is displayed as a receiver operating characteristic curve (area under the curve (AUC) = 80.0%) (Fig. 2).

### 3.2 IL13 Expressing Cases Show Hallmark of Type I IFN Response

**Fig. 3:**
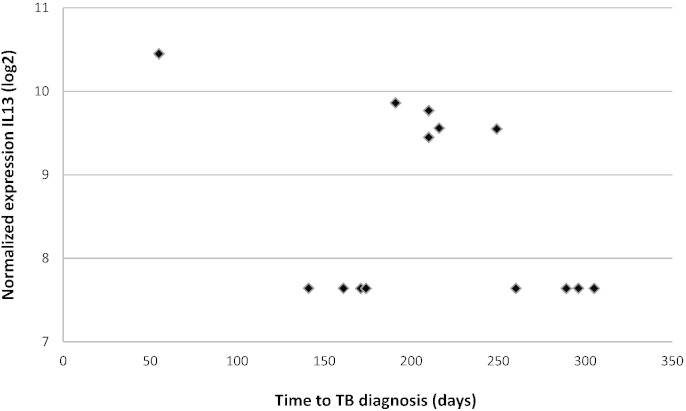
Gene expression and time to diagnosis. GAPDH normalized and log2 transformed gene expression levels of IL13 were plotted against time to TB diagnosis (in days): samples above the threshold of 7.64 represent cases expressing IL13 (IL13^+^). The relationship between gene expression of IL13 and time to TB diagnosis was analyzed using linear regression analysis.

Expression of IL13 was restricted to a subgroup of cases (n = 6/14) and the level of normalized IL13 in that subgroup correlated with time to TB diagnosis (r = − 0.938, P = 0.006) (Fig. 3). The majority (5/6) of IL13^+^ cases were sampled between 190–250 days prior to TB diagnosis and the remaining case close to (on day 55 before) TB diagnosis.

**Fig. 4:**
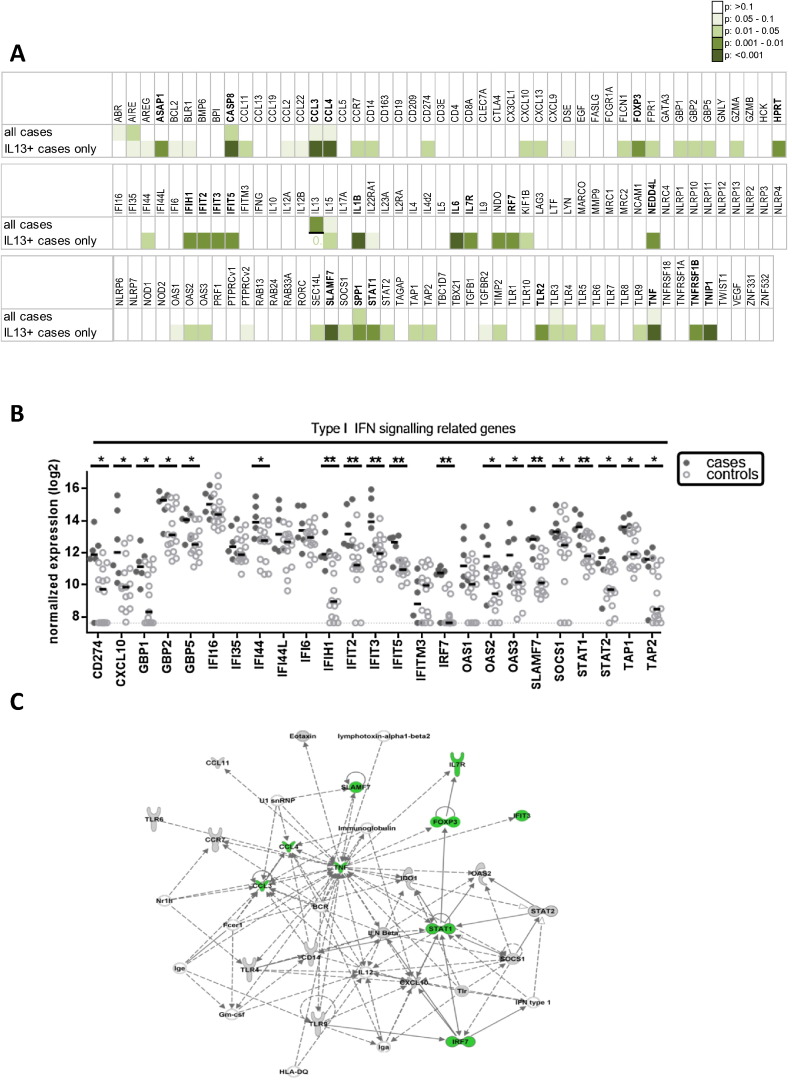
IL13 expressing cases show signature of type I IFN signaling prior to diagnosis. dcRT-MLPA derived gene expression data were reanalyzed using only those cases that expressed IL13 to obtain insights into the pathophysiological process ongoing in these patients-to-be. A. Mann–Whitney testing was performed comparing both the total set of cases with the controls as well as comparing only cases expressing IL13 with all controls; resulting P-values were color coded to visualize genes that were expressed significantly differently. Genes in bold remained significantly differently expressed after multiple testing correction. B. Expression levels of type I interferon related genes are shown only for cases (filled symbols) expressing IL13 and all controls (open circles). Mann–Whitney testing was used to identify differentially expressed genes: *P < 0.05; **P < 0.01. Stars in bold remained significantly different after multiple testing correction. C. Ingenuity based pathway analysis of all genes significantly different between controls and IL13^+^ cases. Genes presented in green remained significantly differently expressed after multiple testing correction.

Since expression of IL13 was the most discriminatory factor according to Lasso regression analysis we reanalyzed gene expression data using only samples of cases expressing IL13 and of all controls to obtain more insights into the pathophysiology of the disease. Focussed analysis of IL13^+^ cases revealed 50 genes that were significantly differently expressed between the study groups (n = 6 cases, n = 15 controls) (Fig. 4A). Interestingly, a considerable number of these genes (n = 19/50) belonged to type I IFN signaling and reached high levels of significance (n = 7/19 with P < 0.01) in the comparison of IL13^+^ cases with controls (Fig. 4B). All genes significantly differently expressed showed increased expression in cases compared to controls. In addition, a large proportion of genes associated with immune activation and inflammation became significant in the comparison of the subgroup of cases expressing IL13 with controls. Indeed, Ingenuity Pathway Analysis on all 50 genes that were significantly differently expressed between IL13^+^ cases and controls revealed a key role for type I IFN signaling within the IL13^+^ cases, suggesting that type I IFN plays a role in TB development in these cases (Fig. 4C). After adjusting for multiple testing using the Benjamini–Hochberg correction 22 of the 50 genes remained significantly differently expressed between cases and controls (presented in bold, Fig. 4A, B).

## 4 Discussion

Identification of individuals at high risk of developing pulmonary TB is not possible with the currently available biomarkers and diagnostic tools. Here we have studied a unique sample set consisting of samples of HIV-infected drug users prior to diagnosis of pulmonary TB, which allowed identification of biomarkers that predict TB development months before clinical diagnosis of TB. To the best of our knowledge this is the first demonstration of biomarkers predictive of TB cases before clinical diagnosis. Application of such biomarkers in particular high risk populations could potentially identify individuals that might benefit from preventive treatment before contagious disease has developed.

The combination of IL13 and AIRE expressions appeared to classify cases versus controls with the highest discriminatory power among our gene sets. The majority of case samples expressing IL13 had been obtained 190–250 days prior to TB diagnosis, suggesting that these combined markers are able to identify individuals that will progress to TB disease within 8 months prior to diagnosis. Screening for such markers could allow early identification and subsequent treatment of individuals at high risk of developing disease, such as homeless, drug users, recent TST converters following contact in low endemic areas or HIV-infected individuals. Identification before the development of contagious TB would help in limiting transmission of infection, and, early treatment of cases could reduce morbidity and minimize complications.

IL13 was expressed in about half of the cases up to 250 days prior to diagnosis. Since it cannot be formally excluded that the other eight cases were not yet infected with *Mtb* (all > 140 days prior to diagnosis) and therefore did not show specific gene expression patterns, we focussed on the IL13 expressing cases to obtain more insights into the pathophysiology of TB. Restricting analysis to the 15 controls and the IL13^+^ cases identified 50 genes that were significantly differently expressed between IL13^+^ cases and controls. Interestingly, many of these differentially expressed genes belong to the type I IFN signature that previous studies have consistently reported to discriminate patients with pulmonary TB at the moment of diagnosis from healthy controls (Berry et al., 2010, Maertzdorf et al., 2011a, Maertzdorf et al., 2011b, Maertzdorf et al., 2012, Ottenhoff et al., 2012b, Cliff et al., 2013). Identifying a similar pattern up to 8 months before TB diagnosis strengthens our findings that it is possible to predict TB months before diagnosis and that genes identified here play a role in the development of TB. This was further supported by the high number of genes that remained significantly associated with subsequent TB disease development after multiple testing correction, indicating that selected genes could be strong predictors for TB development with increasing sample sizes.

Type 1 interferon signaling has been identified not only as a biomarker of TB disease but also as a key player in mycobacterial disease pathogenesis. In leprosy, IFNγ-induced antimicrobial genes were mostly detected in self-healing tuberculoid lesions, whereas type I interferon related genes were preferentially expressed in disseminated and progressive lepromatous lesions (Teles et al., 2013). Excessive type I responses have been linked to TB disease exacerbation, the balance of which is tightly controlled by IL-1 and eicosanoids (Mayer-Barber et al., 2014). In *Mtb* infected mice type I interferon receptor deficiency resulted in reduced pathology due to alterations in early, innate immune responses (Dorhoi et al., 2014). Indeed, limitation of type I interferons promoted bacterial containment, and immunotherapy with drugs that augment the eicosanoid prostaglandin E2, indirectly downregulating type I interferons, prevented death in *Mtb* infected mice (Mayer-Barber et al., 2014). These findings support the pathogenic role of type I interferons in TB.

This study identified human biomarkers with a potential to prospectively identify individuals that develop TB, a finding that may have significant implications for early TB diagnosis and treatment. We do acknowledge that our study sample set is small and that our work only provides a proof-of-concept. A larger sample set as well as longitudinal studies will be required to determine optimal signatures and to determine the relevant time window preceding TB diagnosis. Our data indicate that a predictive signature can be detected in ex vivo blood up to 6–8 months prior to clinical diagnosis and these findings support continuation of the search for predictive biomarkers. Several additional factors have to be considered in interpreting our findings. First, only frozen PBMCs were available for analysis and thus RNA was extracted from (unstimulated) PBMCs, however we expect that fixed whole blood (e.g. PAXgene or Tempus tubes) will give similar results as previous studies on PBMCs and whole blood RNA revealed similar patterns (Joosten et al., 2013). If longitudinal samples from a larger, well defined cohort are available, such as the GC6 cohort (The GC#6–74 Consortium), gene expression analyses should preferably be performed in an unbiased manner, e.g. using RNA sequencing. Secondly, our cohort was restricted to HIV-infected individuals, mostly males, thus future studies should investigate whether our findings are generalizable to HIV-negative individuals and to females. However, biomarkers in HIV-infected individuals found in our study were similar to previously identified markers in HIV-uninfected individuals (Berry et al., 2010, Maertzdorf et al., 2011a, Maertzdorf et al., 2011b, Maertzdorf et al., 2012, Ottenhoff et al., 2012b, Cliff et al., 2013), suggesting that many are common and not specific for HIV-infected TB patients. Thirdly, unfortunately no information on socioeconomic status, alcohol abuse or comorbidities (e.g. other infections, diabetes) on individual basis was registered for this cohort, but we should strongly recommend to assess these parameters in future studies as they might be associated with the risk of progression to TB. Finally, in our cohort timing of TB contact and thus potential time of infection was not defined, nor were routine tests performed to determine infection status of cohort participants (as this was outside the scope of the ACS when it was set up in 1985). We limited our sample set to a time window of 1 year prior to diagnosis as we assumed that most individuals that develop TB will do so within 1–2 years following infection. Still we cannot exclude that some cases were not yet infected at the time of blood sampling. In particular, some cases that were diagnosed more than 8 months after sample collection might not have been infected yet at time of sampling.

The identification of IL13 as a potentially predictive biomarker of TB cases is interesting as Th2 immunity and in particular IL13 have recently been associated with pathology in inflammatory disorders including TB. In murine *Mtb* infection studies IL13 was associated with tuberculosis associated pathology; overexpressing IL13 resulted in enhanced pathology mimicking human TB lesions (Heitmann et al., 2014). Moreover, the expression of IL-13 and the Th2 transcription factor GATA3 by human CD8^+^ T-cells have been associated with several inflammatory disorders involving tissue fibrosis including atopic dermatitis, psoriasis and sclerosis (Medsger et al., 2011, Hijnen et al., 2013, Fuschiotti et al., 2009, Fuschiotti et al., 2013). Also in human allergic asthma, IL-13-producing CD8^+^ T-cells isolated from the lung are increased and associated with airway obstruction (Dakhama et al., 2013). Together these studies suggest that IL-13-producing CD8^+^ T-cells are important players in the development of inflammatory disorders in the lung. mRNA levels for IL13 were also found to be increased in *Mtb* infected human lymph nodes, indicating local IL13 expression in *Mtb* lesions, although the cellular source remained undefined (Rahman et al., 2009). We have also recently identified an unusual subset of human *Mtb* specific CD8^+^ T-cells that produce IL-13 (van Meijgaarden K.E., Haks M.C., Ottenhoff T.H.M., Joosten S.A., submitted), suggesting a contribution of these cells in TB pathogenesis.

In conclusion, we show here that host biomarkers predictive of TB disease development up to 8 months prior to TB diagnosis exist, and can be identified directly ex vivo without antigen specific activation or enrichment steps. Analyses need to be repeated on larger, well defined cohorts with advanced state-of-the-art methods.

## Role of the Funding Source

The funding sources had no role in study design, data collection, data analysis, data interpretation, or writing of the manuscript. The corresponding authors had full access to all data and had the final responsibility for the decision to submit for publication.

## Author Contributions

RS, SAJ, THMO, MWB and MS conceived and designed the study. MCH developed the MLPA probes and made them available. SAJ and MCH supervised laboratory analysis. RS, MFSL, SAJ, THMO, MWB, and STK undertook, supervised, or monitored the study. RS and SAJ obtained and analyzed the data. EWZ did the statistical analysis. RS and SAJ interpreted the data, designed figures and drafted the manuscript. All authors contributed to the refinement of the study report and approved the final version.

## Declaration of Interests

We declare that we have no competing interests.

## Figures and Tables

**Table 1:** Demographic and clinical characteristics of HIV-infected individuals participating in the Amsterdam Cohort Studies included in the study.

	Cases n (%)	Cases IL13 pos n (%)	Cases IL13 neg n (%)	Controls n (%)	P-value^1^	P-value^2^	P-value^3^
Total	14	6	8	15			
Sex							
Male	12 (86)	4 (67)	8 (100)	13 (87)	1.000^a^	0.544^a^	0.165^a^
Female	2 (14)	2 (33)	0 (0)	2 (13)			

Age							
Years; median (IQR)	38 (33–41)	37 (31–40)	39 (33–44)	37 (35–46)	0.847^b^	0.622^b^	0.414^b^
25–34	5 (36)	2 (33)	3 (38)	3 (20)	0.632^a^	0.534^a^	0.627^a^
35–44	7 (50)	4 (67)	3 (38)	8 (53)			
≥ 45	2 (14)	0 (0)	2 (25)	4 (27)			

TB classification							
PTB	8 (57)	5 (83)	3 (38)				0.096^a^
EPTB	2 (14)	1 (17)	1 (13)				
PTB + EPTB	4 (29)	0 (0)	4 (50)				

Sputum smear status							
Smear-positive	7 (50)	3 (50)	4 (50)				1.000^a^
Smear-negative	5 (36)	2 (33)	3 (38)				
NA⁎	2 (14)	1 (17)	1 (13)				

Culture status							
Positive	12 (86)	5 (83)	7 (88)				1.000^a^
Negative⁎⁎	2 (14)	1 (17)	1 (13)				

CD4 cell count							
Cells per mm^3^; median (IQR)	270 (168–400)	315 (260–603)	190 (138–325)	340 (220–430)	0.354 b	0.622^b^	0.081^b^

Viral load⁎⁎⁎							
By p24 ELISA (pg/ml); median (IQR) (n = 17)	18 (0–101) (n = 10)	32 (14–154) (n = 5)	0 (0–56) (n = 5)	0 (0–112) (n = 7)	0.417^b^	0.106^b^	0.095^b^
By RNA (copies/ml); median (IQR) (n = 12)	13,000 (2863–191,250) (n = 4)	11,000 (n = 1)^c^	150, 15,000, 250,000 (n = 3)^c^	8300 (400–62,905) (n = 8)	0.932^b^	1.000^b^	1.000^b^

Extent of lung damage on chest X-ray							
Mild	4 (29)	2 (33)	2 (25)				0.571^a^
Moderate	6 (43)	3 (50)	3 (38)				
Extensive	2 (14)	0 (0)	2 (25)				
NA⁎	2 (14)	1 (17)	1 (13)				

Calendar period of sample (year)							
1985–1993	5 (36)	3 (50)	2 (25)	4 (27)	0.791 a	0.311^a^	0.627^a^
1994–2002	7 (50)	3 (50)	4 (50)	7 (47)			
≥ 2003	2 (14)	0 (0)	2 (25)	4 (27)			

P-values represent:

1 Comparison between all TB cases and all controls.

2 Comparison between IL13 positive cases and all controls.

3 Comparison between IL13 positive cases and IL13 negative cases.

a Tested by Fisher's Exact Test.

b Tested by Mann–Whitney U test (using exact significance).

c Numbers too small to calculate median and IQR.

⁎ NA = these represent the 2 TB cases with EPTB; this category was excluded from analysis comparing IL13 positive and IL13 negative cases.

⁎⁎ Both culture negative TB cases responded to treatment.

⁎⁎⁎ A p24 ELISA was done for all cases and controls sampled before 1997, and a RNA test was done for all cases and controls sampled thereafter.
